# A Combined High and Low Cycle Fatigue Model for Life Prediction of Turbine Blades

**DOI:** 10.3390/ma10070698

**Published:** 2017-06-26

**Authors:** Shun-Peng Zhu, Peng Yue, Zheng-Yong Yu, Qingyuan Wang

**Affiliations:** 1 Center for System Reliability & Safety, University of Electronic Science and Technology of China, Chengdu 611731, China; zspeng2007@uestc.edu.cn (S.-P.Z.); yuepeng0317@163.com (P.Y.); yuzhengyongyong@126.com (Z.-Y.Y.); 2 Key Laboratory of Deep Earth Science and Engineering, Ministry of Education, Sichuan University, Chengdu 610065, China; 3 School of Architecture and Civil Engineering, Chengdu University, Chengdu 610106, China

**Keywords:** turbine blade, combined cycle fatigue, damage accumulation, life prediction, HCF, LCF

## Abstract

Combined high and low cycle fatigue (CCF) generally induces the failure of aircraft gas turbine attachments. Based on the aero-engine load spectrum, accurate assessment of fatigue damage due to the interaction of high cycle fatigue (HCF) resulting from high frequency vibrations and low cycle fatigue (LCF) from ground-air-ground engine cycles is of critical importance for ensuring structural integrity of engine components, like turbine blades. In this paper, the influence of combined damage accumulation on the expected CCF life are investigated for turbine blades. The CCF behavior of a turbine blade is usually studied by testing with four load-controlled parameters, including high cycle stress amplitude and frequency, and low cycle stress amplitude and frequency. According to this, a new damage accumulation model is proposed based on Miner’s rule to consider the coupled damage due to HCF-LCF interaction by introducing the four load parameters. Five experimental datasets of turbine blade alloys and turbine blades were introduced for model validation and comparison between the proposed Miner, Manson-Halford, and Trufyakov-Kovalchuk models. Results show that the proposed model provides more accurate predictions than others with lower mean and standard deviation values of model prediction errors.

## 1. Introduction

Modern aircraft engines tend to meet the requirements for high thrust-to-weight ratio and high reliability [1,2]. As one of the fracture critical components of an aero-engine, the turbine blade is often subjected to high mechanical stresses resulting from centrifugal force, vibratory and flexural stresses, and thermal stresses under complex engine operational conditions [3]. Different from integral blade rotors, this is a potential source of trouble in the blade-disc fixture, which is usually of the dove-tail, hammer type or fir tree type. All these concepts ensure that the blades are tightly fitted on the disc through considering the effect of finishing on the blade strength and life [4,5,6]. In particular, the structural integrity of a turbine blade can be threatened by two or more damage mechanisms: (a) fatigue, including thermal fatigue, multiaxial fatigue (high cycle fatigue (HCF)/low cycle fatigue (LCF)); (b) creep; (c) corrosion; and (d) foreign object damage and oxidation [7,8,9,10,11,12,13,14,15]. It is worth noting that fatigue strength of a turbine blade is often greatly reduced during the engine’s operation, even giving rise to its premature failure [16,17,18,19,20]. In addition, improving the fatigue resistance of the turbine blade is critical to increasing the gas temperature for high thrust-to-weight ratio [21]. Typically, aircraft gas turbine blades are subjected to a complex interaction of low-frequency centrifugal forces with large amplitude superimposed with small amplitude, high-frequency vibrations simultaneously under engine operating conditions [22,23]. The amplitude of vibration loads is relatively small even below the endurance limit of the turbine blade and, therefore, vibrations generate a HCF failure. In contrast, low-frequency centrifugal forces lead to a LCF failure. Among them, LCF loads result from the takeoff-cruise to landing engine cycles, HCF loads result from the aerodynamically in-flight vibrations [24]. Thus, both of HCF and LCF usually induce failure of turbine blades, which is named as combined high and low cycle fatigue (combined cycle fatigue (CCF) in this analysis) of aircraft gas turbine blades. From the viewpoint of turbine blade design, the effect of superimposing LCF to HCF needs to be considered through modeling the combined damage accumulation on the expected fatigue strength behavior by CCF tests [25].

Since the fatigue problems under combined cycle loadings were noticed by Fuchs et al. [26], recently, increasing attention has been paid for the CCF failure analysis. Namjoshi et al. [27] calculated the damage fraction from LCF and HCF components of combined cycling under fretting fatigue loadings. Oakley et al. [28] presented a life prediction model under CCF conditions by considering foreign object damage. Schweizer et al. [29] put forward a mechanism-based model to describe the micro-cracks evolution behavior under CCF loadings, and pointed out that crack growth rates were accelerated under HCF-LCF interaction. Stanzl-Tschegg et al. [30] conducted variable amplitude fatigue tests using high-frequency cycles superimposed on low-frequency loads. The experimental result indicated that fatigue lives under combined variable amplitude loading were much shorter than those under constant amplitude loading. Meanwhile, various models [31,32,33,34,35,36,37] have been presented for CCF life prediction. Among them, through characterizing the effects of high cycle stress amplitude and low cycle stress amplitude on combined fatigue damage, Zheng et al. [36] developed a CCF life prediction model based on the exponential decay law. Karunananda et al. [37] estimated the combined fatigue life of structures by introducing a new damage indicator and strain-life fatigue curve.

From the viewpoint of practical application, the linear damage rule (also named as Miner’s rule) [38] has been frequently used to assess cyclic CCF damage because of its simplicity, however, it ignored the effects of load interactions on CCF damage modeling and life estimation. The coupled damage caused by HCF-LCF interaction is rarely considered in current CCF life prediction methods. Moreover, load interactions exert significant effects on the combined cycle fatigue life. Based on the observation, a CCF life prediction model is proposed to simultaneously take into account the coupled damage and the influence of load conditions on CCF life. 

The rest of this paper is organized as follows: Section 2 is devoted to a generic HCF design and verification process and commonly-used methods for CCF life prediction, including both linear and nonlinear damage accumulation models; Section 3 elaborates a new CCF life prediction model by considering the coupled damage due to HCF-LCF interaction. Then the proposed model is verified with five experimental datasets of turbine blade alloys and turbine blades comparing with three other models in Section 4; Finally, Section 5 gives a conclusion to this paper. 

## 2. HCF-LCF Interaction

### 2.1. HCF Design and Verification

HCF is a generic problem for aero-engine components, which may be initiated by, or interact with, other damage modes, such as creep, thermal fatigue, and LCF in engine components, like turbine blades. Among them, HCF-LCF interaction usually leads to the aforementioned CCF failure. In order to reduce HCF risk and occurrences, a comprehensive procedure which can improve the current design and verification system is needed, especially the prediction methods for assessing HCF drivers and component responses. In general, different sources of HCF damage in aero-engines can be classified as follows: (i) aerodynamic excitations caused by engine flow path pressure perturbations; (ii) mechanical vibrations caused by rotor imbalance; and (iii) airfoil flutters caused by aeromechanical instability. 

**Figure 1:**
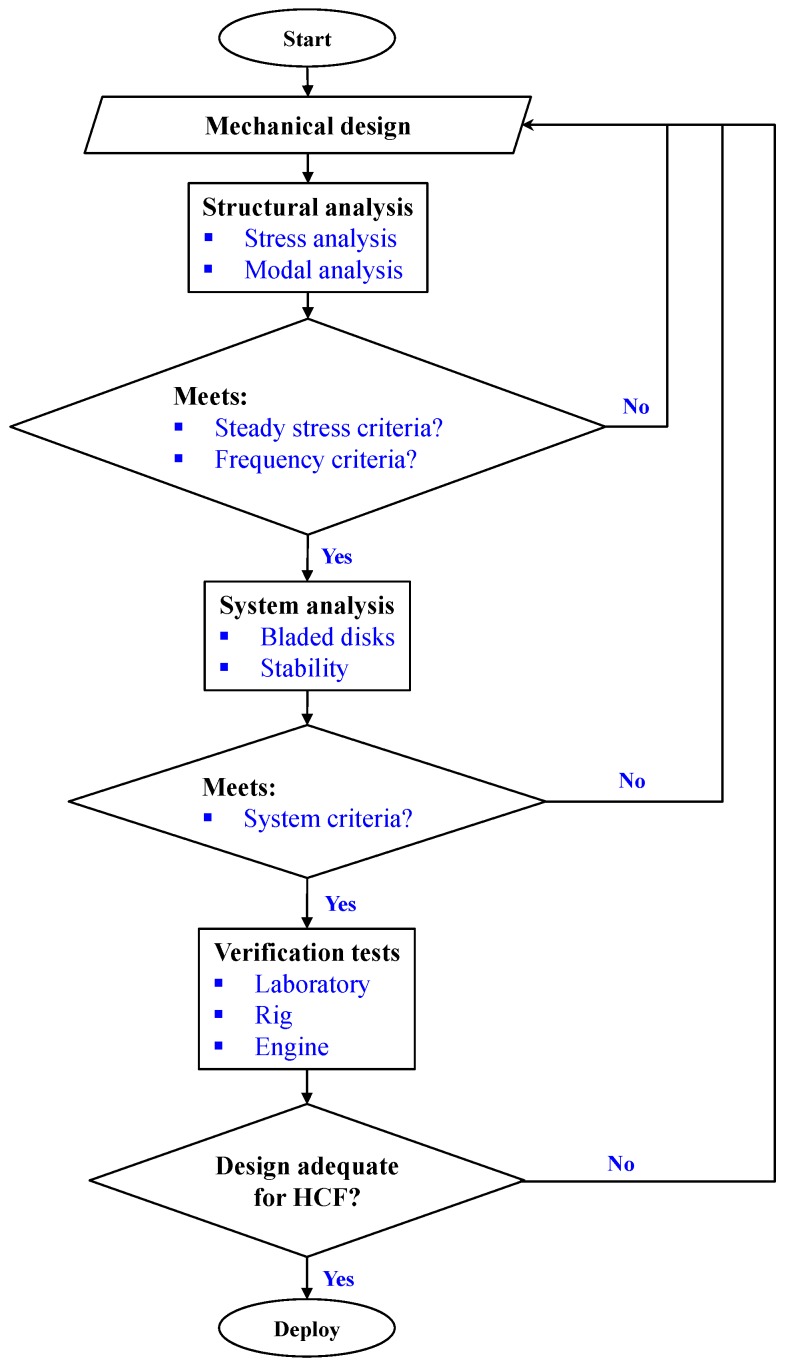
A generic procedure for HCF design and verification.

**Figure 2:**
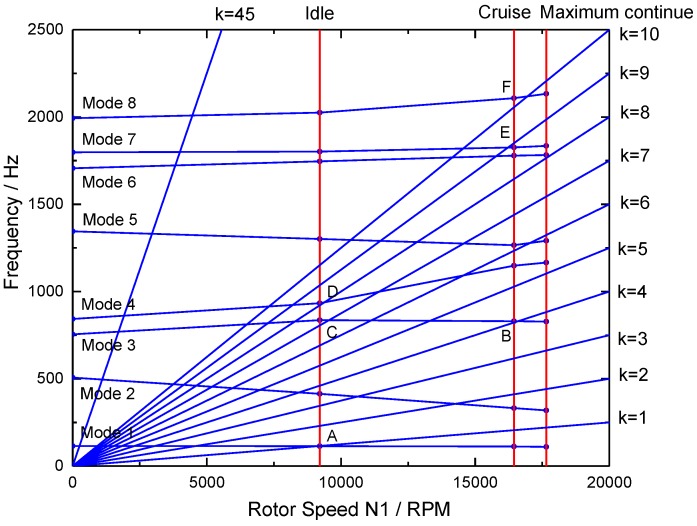
Typical Campbell diagram of a high pressure turbine blade showing selected crossings.

For turbine blades, HCF capability assessment generally consists of two steps based on finite element (FE) analysis: a stress analysis to assess mean or steady stresses, and then a structural dynamics analysis to obtain resonant frequencies and mode shapes. Figure 1 outlines a generic process for HCF risk analysis and design of aircraft gas turbine blades. Following this process, the HCF design configuration is iterated until it meets the steady stress limit criteria, and the engine component response frequencies avoid matching frequencies of known strong excitations during the engine operations. As an example, predicted resonances of a high pressure turbine blade are compared with these integral order engine excitations on a typical Campbell diagram for three typical flight missions: idle, maximum continue and cruise (see Figure 2). Turbine blade designs may result in unavoidable crossings of resonant modes or presumed weaker drivers. 

Once the design configuration in Figure 1 is judged acceptable, the engine components, like turbine blades, are manufactured and subjected to both laboratory and engine verification testing [5,6], including laboratory measurement of response frequencies, mode shapes, normalized stresses, and so on. In the gas turbine industry, one of the most empirical aspects of the current approach to HCF is the material/component capability assessment without considering the effect of interacting failure modes like LCF, today which are almost entirely ignored during the turbine blade life and reliability predictions. 

### 2.2. CCF Life Prediction

**Figure 3:**
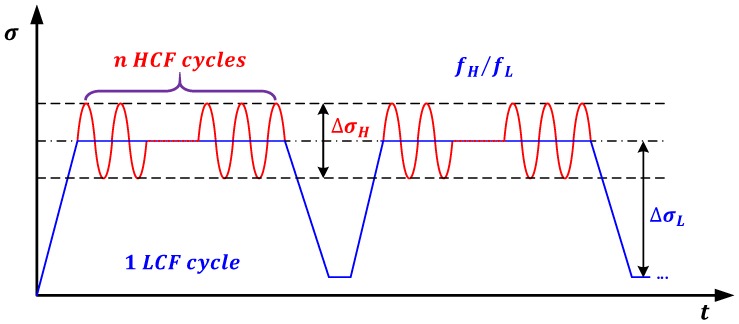
A load spectrum for CCF analysis.

As aforementioned, the CCF life prediction issue is still one of the puzzles that need to be explored for aircraft gas turbine blades. Accordingly, studies by [21,23,31,32,33,34,36] introduced a typical load spectrum to simulate operating conditions of actual gas turbine attachments in laboratory, and analyze the CCF behavior through testing of materials and/or components as shown in Figure 3. Specifically, this load spectrum can be mainly characterized by four load parameters, namely, the high cycle stress range $∆\sigma_{H}$ and frequency $f_{H}$, the low cycle stress range $∆\sigma_{L}$ and frequency $f_{L}$. 

Based on fatigue damage accumulation theory, the Coffin-Manson equation is commonly used together with the Miner’s rule to estimate the CCF life and combined damage accumulation of HCF and LCF. In general, fatigue damage accumulation under variable amplitude loadings can be calculated by using the linear damage rule [38]:

(1) $$D=\overset{k}{\underset{i=1}{\sum }}\frac{n_{i}}{N_{Ri}}$$

where $n_{i}$ is the number of loading cycles at the stress level $\sigma_{i}$, $N_{Ri}$ is the number of cycles to failure at $\sigma_{i}$, D is the cumulative damage, k is the number of stress levels. 

Through the extension of Miner’s rule to the CCF loading cases, a life fraction equation for combined cycle fatigue damage accumulation can be similarly derived as follows [32,39,40]:

(2) $$D_{i}=\sum^{\text{​}}\frac{n_{i}}{N_{Ri}}=\frac{n_{HCF,i}}{N_{HCF,i}}+\frac{1}{N_{LCF,i}}$$

where $n_{HCF,i}$, $N_{HCF,i}$, and $N_{LCF,i}$ are the number of loading cycles of HCF, the number of cycles to failure of HCF, and the number of cycles to failure of LCF at the given combined cycle block for the ith level, respectively.

Assuming that fatigue failure occurs once the cumulative damage is equal to the critical value, such as one. Then CCF life can be calculated by using Equation (2), as follows, when high cycle stress amplitude and low cycle stress amplitude of the CCF load spectrum are constant [32,39,40]:

(3) $$N=\frac{1+n}{\frac{n_{HCF}}{N_{HCF}}+\frac{1}{N_{LCF}}}$$

where $N_{HCF}$ is the number of cycles to failure of HCF, $N_{LCF}$ is the number of cycles to failure of LCF, n is the ratio of high and low cycle stress frequency.

Using Equation (2), the Miner’s rule calculated the HCF and LCF damage individually. In other words, fatigue damage is considered for pure HCF and LCF loads, it assesses CCF life by simple superposition of these two components of damage independently.

From a nonlinear damage accumulation point of view, Manson and Halford [41] developed a nonlinear model for CCF life prediction based on the Miner’s rule:

(4) $$\{\begin{array}{c} D_{i}={\left(\frac{n_{i}}{N_{Ri}}\right)}^{q_{i}}\\ q_{i}=BN_{Ri}^{\beta } \end{array}$$

where $q_{i}$ is the damage exponent, and B and β are material constants determined from experiments. 

Similarly, based on uniaxial loading and bending fatigue tests, Trufyakov and Kovalchuk [35] analyzed the effect of several metal materials under different frequencies and ratios of high and low cycle stresses on combined cycle fatigue life, which indicated that a linear relationship remains between the two-frequency loading coefficient and the ratios of high and low cycle stresses with a change in the frequency ratios and strength properties, and presented a life prediction model under two-frequency loadings as:

(5) $$N=\left(1+n\right)N_{LCF}{\left(\frac{1}{n}\right)}^{\gamma^{\sigma_{a,HCF}/\sigma_{a,LCF}}}$$

where γ is material constant, $\sigma_{a,HCF}$ is the high cycle stress amplitude, $\sigma_{a,LCF}$ is the low cycle stress amplitude. 

Note from [29,30,36] that coupled damage due to the HCF-LCF interaction exists under CCF conditions in addition to pure HCF and LCF damages. Moreover, HCF and LCF interaction has shown great influences on CCF life and even accelerates crack growth process of materials. This is because of larger interaction damage produced by the larger low cycle stresses, and the interaction damage increases with high cycle stress amplitude. Since many HCF-risk engine components and locations are exposed to some LCF loadings, the current approaches do not adequately address the interaction of HCF with other damage modes. Hence, a more accurate CCF life prediction method is expected for practical applications.

## 3. Model Development

When the stress amplitude of load spectrum for CCF is under constant amplitude loading, the cumulative damage for N loading cycle blocks based on the Miner’s rule under CCF loadings is defined as:

(6) $$D=N\left(\frac{n_{HCF}}{N_{HCF}}+\frac{1}{N_{LCF}}\right)$$

In this analysis, the combined cumulative damage is calculated by the sum of HCF damage, LCF damage, and high-low cycle coupled damage. In other words, fatigue damage caused by the mutual coupled effect derived from CCF loads, apart from the damage caused by pure HCF and LCF loads. Based on this, the total cumulative damage of N loading cycle blocks can be derived as:

(7) $$D=N\left(\frac{n_{HCF}}{N_{HCF}}+\frac{1}{N_{LCF}}+D_{c}\right)$$

where $D_{c}$ is the coupled damage caused by the combined high and low cycle stresses. 

According to the CCF load spectrum in Figure 3, the ratio of high and low cycle stress range, α, is given by:

(8) $$\alpha =\frac{∆\sigma_{H}}{∆\sigma_{L}}$$

The ratio of high and low cycle stress frequency, n, is:

(9) $$n=\frac{f_{H}}{f_{L}}$$

**Figure 4:**
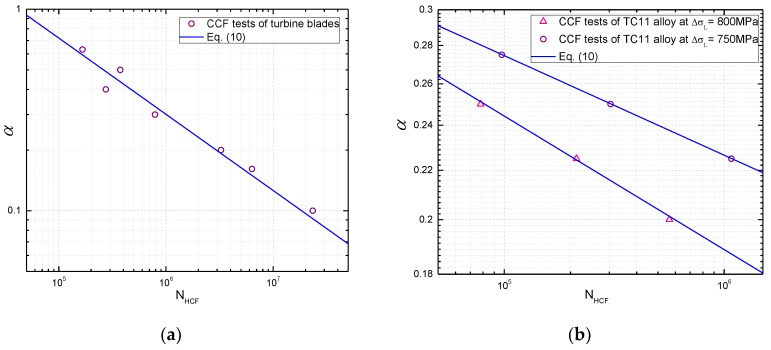
Fitted curves between α and HCF cycles based on CCF tests of (**a**) turbine blades [42] and (**b**) TC11 alloys [32].

In order to quantify the relationship between the coupled damage and CCF life, four load parameters in the load spectrum are introduced to model the coupled damage. Note from the experimental results that certain relationships can be obtained between the HCF cycles and the ratio of high and low cycle stress amplitude α, as shown in Figure 4. Yan et al. [42] designed a CCF test procedure for turbine blades to study the relationship between HCF cycles and vibration stress amplitudes. The ratio of high and low cycle stress amplitude α is calculated in this paper, and the relationships between α and HCF cycles (logarithm) are presented for turbine blades as shown in Figure 4a. Similarly, a linear relationship for TC11 alloy can be obtained, as shown in Figure 4b according to the experimental data in [32].

As it can be seen from the CCF load spectrum, there are (n+1) cycles including one LCF cycle and n HCF cycles in a combined cycle block. Moreover, note from [32,42] that the ratio of high and low cycle stress amplitude and the number of cycles to failure of HCF are approximately linear in a single logarithmic coordinate. Then an expression can be obtained by

(10) $$\alpha =alogN_{HCF}+b$$

where a and b are material constants.

From the view point of loading waveform under CCF in which the four load parameters have shown significant effects on its life behavior, the damage interaction of n HCF cycles with the one LCF cycle in one combined cycle block is considered. Combining with the relationship between the ratio of high and low cycle stress amplitude and HCF life, then a new coupled damage indicator is defined to quantify the relationship between load parameters and coupled damage according to the HCF life $N_{HCF}$ by using the high-low cycle stress range ratio α and high-low cycle stress frequency ratio n as,

(11) $$D_{c}=\frac{1}{\left(n+1\right)log{\left(N_{HCF}\right)}^{\alpha }}$$

Thus, the total fatigue damage by using the life fraction model under CCF can be derived according to Equation (7) as follows 

(12) $$D=N\left(\frac{n}{N_{HCF}}+\frac{1}{N_{LCF}}+\frac{1}{\left(n+1\right)log{\left(N_{HCF}\right)}^{\alpha }}\right)$$

When the cumulative damage reaches the critical one, the number of combined cycle blocks $N_{B}$ can be calculated by 

(13) $$N_{B}={\left(\frac{n}{N_{HCF}}+\frac{1}{N_{LCF}}+\frac{1}{\left(n+1\right)log{\left(N_{HCF}\right)}^{\alpha }}\right)}^{-1}$$

Using Equation (13), the number of combined cycle fatigue to failure is obtained by $N_{f}=\left(n+1\right)N_{B}$.

## 4. Experimental Validation

### 4.1. Model Validation for Turbine Blade Alloys

In order to verify the proposed life fraction model in Equation (13), four experimental datasets of turbine blade alloys, including TC11 [32], Ti-6Al-4V [34], Al 2024-T3 [31], and DZ22 alloy [43], are introduced for model validation under CCF loadings with different high-low cycle stress amplitude ratios and high-low cycle stress frequency ratios. Moreover, model predictions by the proposed model, Miner’s rule, Manson-Halford model, and Trufyakov-Kovalchuk model are compared with experimental results under CCF loadings.

**Figure 5:**
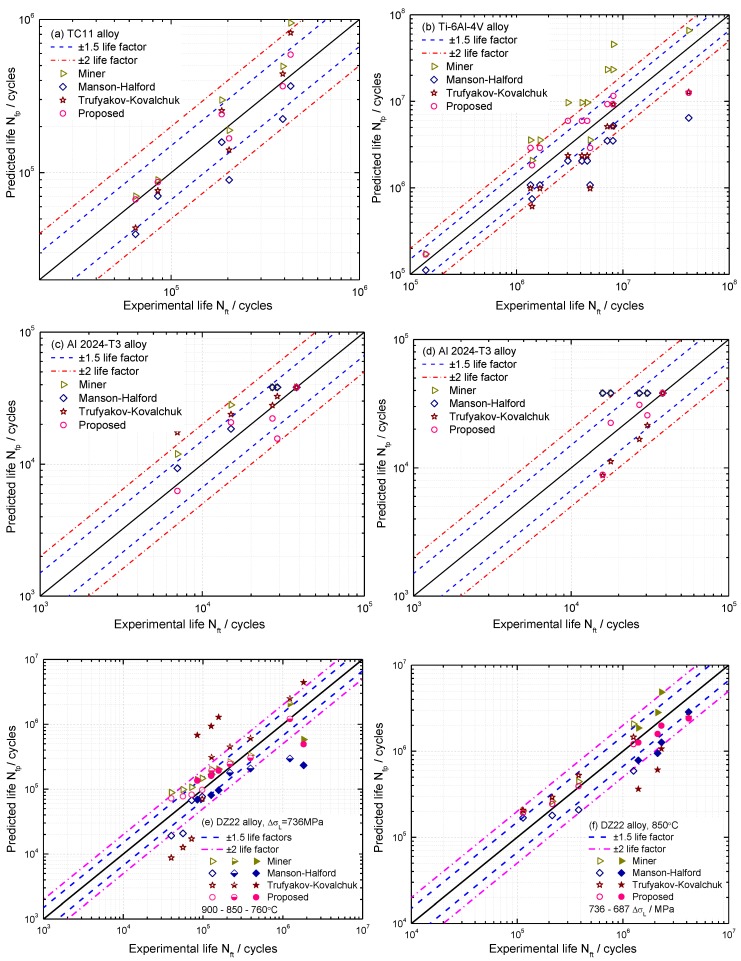
Comparison between experimental results and model predictions of (**a**) TC11; (**b**) Ti-6Al-4V, (**c**) Al 2024-T3 for different α ; (**d**) Al 2024-T3 for different n; (**e**) DZ22 alloy for different temperatures and (**f**) DZ22 alloy for different $∆\sigma_{L}$.

In this analysis, fatigue tests were conducted under pure LCF, pure HCF, and CCF loadings. Accordingly, fatigue performance of TC11 alloy was investigated according to the LCF, HCF, and CCF tests in [32]. For LCF tests of TC11, the loading pattern was the trapezoidal wave with a frequency of 1 Hz and a stress ratio of zero, as shown in Figure 3. For HCF tests, the load was purely applied by the sinusoidal wave with a frequency of 12 Hz and 16 Hz changing with the load amplitude. Under CCF loading conditions, the ratio of high and low cycle stress frequency is 1000. More details on experimental procedure under study can be referred to [31,32,34,43]. For the proposed model, the ratio of high and low cycle stress amplitude is obtained firstly using Equation (8). Then, fatigue life is calculated using Equation (13) and life prediction results and life factors are presented in Figure 5. For the Trufyakov-Kovalchuk model, the material constant γ is suggested as 1.3–1.7 for different materials. In this paper, γ is 1.6 for titanium and Nickel base DZ22 alloys, while it is 1.3 for aluminum alloy, according to [32,35]. Figure 5 shows that the predictions by the Miner’s rule tend to be overestimated. Compared with the Miner’s rule, the nonlinear damage accumulation models including the Manson-Halford and Trufyakov-Kovalchuk models provide more accurate life predictions. It is worth noting that the proposed model provides the most accurate predictions than others with a tighter dispersion. This is because it not only considers the fatigue damage caused by HCF and LCF loads, but also includes the coupled damage caused by the combined cycle fatigue loads. In other words, the ratio of high and low stress amplitude and the ratio of high-low cycle stress frequency are important impact factors for CCF predictions. 

Moreover, the life prediction error has shown an increasing trend along with the cumulative damage of CCF caused by HCF loads for the four materials. Thus, the other three models referred to in this paper cannot accurately model the fatigue behavior of materials under CCF loadings.

For different high-low cycle stress amplitude ratios, nearly all the data points for these four alloys are predicted within the region of ±2 life factors by the proposed model. Specifically, all predictions lie within the range of ±1.5 life factors for TC11 alloy, seven of the 12 predicted results for Ti-6Al-4V alloy, four of the five predicted results for Al 2024-T3 alloy, and 14 of the 20 predicted results for DZ22 alloy. In Figure 5, the Manson-Halford model tends to underestimate the CCF life for TC11 and Ti-6Al-4V alloys and overestimates for Al 2024-T3 alloy. For Ti-6Al-4V alloy, the Miner’s rule provides non-conservative predictions, whereas conservative predictions are provided by the Trufyakov-Kovalchuk model. For different high-low cycle stress frequency ratios, all the model predictions by both of the proposed and Trufyakov-Kovalchuk models are within the range of ±2 life factors. 

According to model predictions in Figure 5, a statistical analysis was also conducted to quantify the model prediction errors $P_{error}$ for model comparison [44,45]. The model prediction error can be obtained by the difference between the logarithmic model prediction and logarithmic experimental result:

(14) $$P_{error}=log_{10}\left(N_{fp}\right)-log_{10}\left(N_{ft}\right)$$

where $N_{fp}$ is the model predicted life, and $N_{ft}$ is the experimental life.

**Figure 6:**
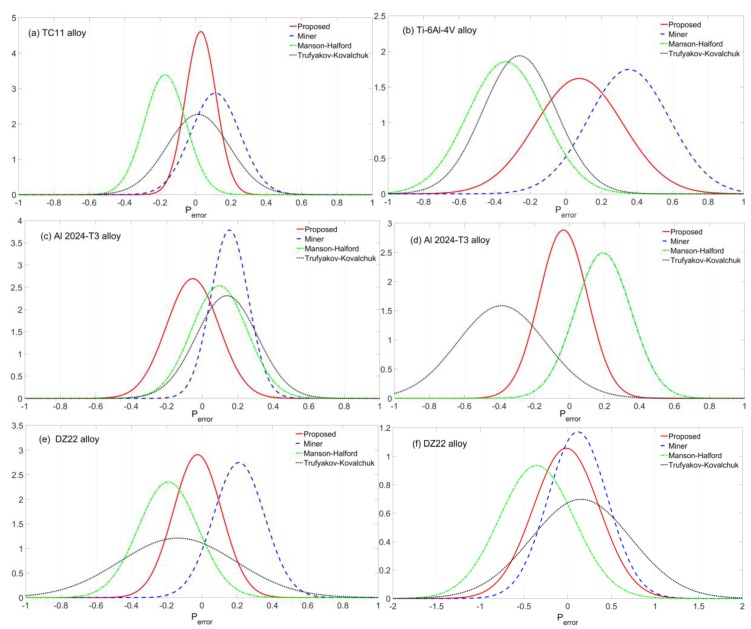
Model prediction errors of (**a**) TC11; (**b**) Ti-6Al-4V; (**c**) Al 2024-T3 for different α; (**d**) Al 2024-T3 for different n; (**e**) DZ22 alloy for different temperatures and (**f**) DZ22 alloy for different $∆\sigma_{L}$.

Using Equation (14), the model prediction errors of the proposed, Miner, Manson-Halford, and Trufyakov-Kovalchuk models under CCF are compared as shown in Figure 6, model prediction errors under different high-low cycle stress amplitude ratios are presented in Figure 6a–c, Figure 6d shows different high-low cycle stress frequency ratios, and Figure 6e,f shows the DZ22 alloy under different temperatures and low cycle stress amplitudes, respectively.

For Al 2024-T3 alloy as shown in Figure 6d, the predicted lives by the Manson-Halford model are the same as that of Miner’s rule, since the fatigue damage of HCF loads is relatively small for these two models. In other words, CCF life is equal to the life under pure LCF. Thus, these two predictions by the Miner’s rule and Manson-Halford model are overlapping as shown in Figure 6d. 

As noted from the statistical analysis of the four models in Figure 6, considering the overall performance, the proposed model provides more accurate predictions than others with lower mean and standard deviation (SD) values of model prediction errors. For the Miner’s rule, the fatigue lives are overestimated with positive mean errors for these four alloys. Moreover, the model prediction errors have shown a growing trend along with the increasing of high-low cycle stress amplitude ratio. 

### 4.2. Model Validation for Turbine Blades

As it can be seen from life prediction results and error analysis of turbine blade alloys, the proposed model has achieved satisfactory predictions for bar specimens under CCF loadings. In this section, CCF life prediction of turbine blades are carried out to verify the accuracy of the proposed model. As aforementioned, the CCF tests of turbine blades are conducted under the LCF and HCF loads according to the load spectrum of CCF in Figure 3. For this load spectrum, the engine rotor speeds were monitored from real flight missions, including 0-maximum continue-0, idle-maximum continue-idle, cruise-maximum continue-cruise, and then the centrifugal force of the turbine blade can be calculated, while the high cycle stress amplitude varies with different flight missions. In order to carry out CCF tests of turbine blades, a new experimental scheme has been designed and developed using a Ferris Wheel (a LCF tester) to simulate the LCF load overlapping high cycle vibration load applied by electromagnetic exciter at elevated temperature [23,42]. During the CCF tests, firstly, the low cycle stress amplitude was designed according to the centrifugal force, which was approximated in the CCF tests by a trapezoidal wave presented in Figure 3; the sinusoidal wave results from in-flight vibrations which give rise to HCF shown in the load spectrum of the CCF; based on this, the experimental lives of turbine blades can be obtained under different high-frequency vibration loads by superimposed HCF cycles upon the dwell portion of LCF cycles. 

**Figure 7:**
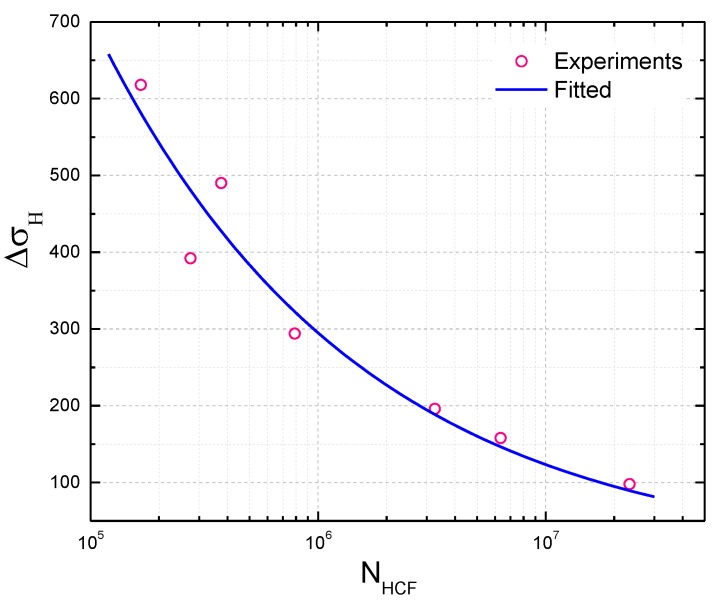
Fitted $∆\sigma_{H}-N_{HCF}$ curve of the turbine blade.

**Figure 8:**
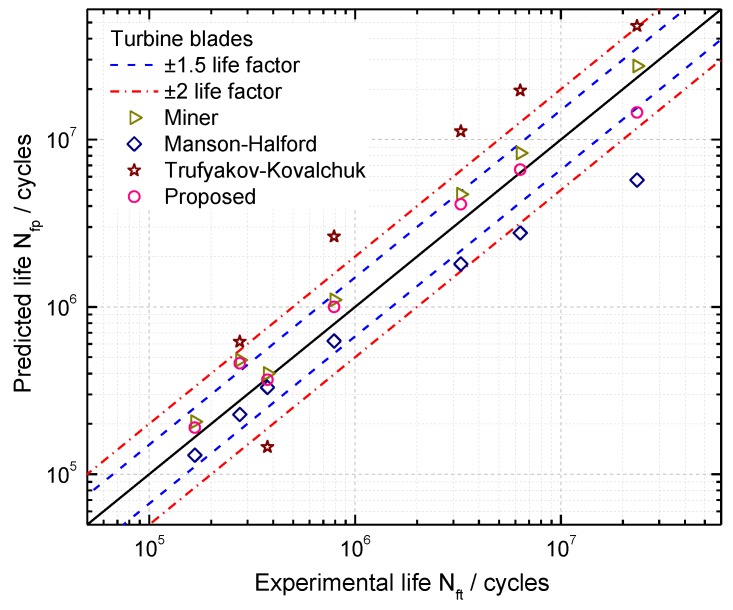
CCF life prediction results of turbine blades.

**Figure 9:**
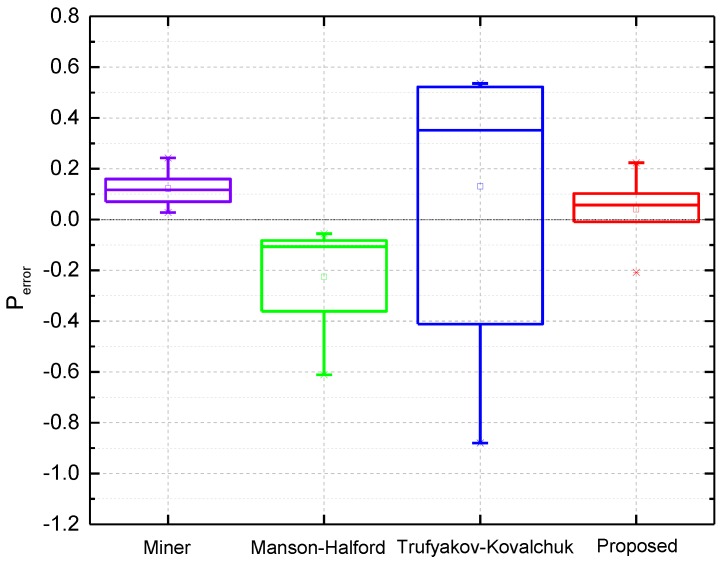
Model prediction errors of turbine blades.

In this paper, combined cycle fatigue test data of seven turbine blades in [42] are introduced for model validation. Based on the pure LCF tests of turbine blades, the LCF life of turbine blades is $N_{LCF}=40,530$ cycles, the ratio of high-low cycle stress frequency is n=5000. The CCF test loads and results are listed in Table 1. In order to calculate the CCF life of the turbine blade, its *S-N* curve can be firstly fitted from the number of cycles to failure of HCF under the given stress amplitudes, which is presented in Figure 7. Then CCF life of turbine blades can be estimated by using Equation (13). The proposed model, Miner’s rule, Manson-Halford and Trufyakov-Kovalchuk models are used for estimating CCF lives of these turbine blades, prediction results are presented in Figure 8. Due to small sample tests of turbine blades in this analysis, box plots on the model prediction errors are created for model comparisons, as shown in Figure 9. 

As it can be seen from Figure 8 and Figure 9, the prediction results by the Manson-Halford model tend to be underestimated. However, both of the proposed model and Miner’s rule provide more accurate predictions than others, which are within the ±1.5 scatter factor. For the Trufyakov-Kovalchuk model, it predicts the blade lives with a larger scatter than others compared with the experimental ones, since it introduced an extra fitted material constant γ to account for the interaction of high and low cycle stress amplitude, which limits the further application of this model under different loading conditions, especially under limited test data conditions. 

## 5. Conclusions

In the present paper, a generic procedure for HCF design and verification is explored, then fatigue damage accumulation methods for CCF life prediction are analyzed for turbine blades. Experimental data of four turbine blade alloys and turbine blades are introduced for model validation and comparison with the Miner, Manson-Halford, and Trufyakov-Kovalchuk models. The conclusions can be drawn as follows.

(1) Based on Miner’s rule, a new life fraction model is proposed for CCF life prediction by introducing a coupled damage component, which addressed the contribution of HCF to the growth of LCF cracks and can be calculated from a function of the four load parameters.
(2) For TC11, Ti-6Al-4V, Al 2024-T3, and DZ22 alloys, nearly all the data points are predicted within a scatter band of ±2 by the proposed model, and 35 out of 48 cyclic lives are within a ±1.5 scatter factor, whereas the Manson-Halford model underestimates the CCF life. The Miner’s rule overestimates the CCF life of the four turbine blade alloys. For the turbine blades, both of the proposed model and Miner’s rule provide reasonably acceptable correlations with tested lives within ±1.5 scatter factor. Considering the overall prediction performance of each model mentioned above, statistical analysis of model prediction errors has shown that the proposed one yields more accurate CCF life predictions than others with lower mean and SDs of model prediction errors.

## Figures and Tables

**Table 1:** CCF test data of turbine blades under different stress levels.

No.	Amplitude of Vibration at Blade-Tip/mm	$∆\sigma_{H}$/MPa	CCF Life $N_{ft}$	
			$N_{LCF,t}$/Cycles	$N_{HCF,t}$/10^3^ Cycles
1	0.5	98	4676	23380
2	0.8	158	1268	6340
3	1.0	196	652	3260
4	1.5	294	158	790
5	2.0	392	55	275
6	2.5	490	75	375
7	3.2	618	26	166.4

## Nomenclature

n	Ratio of high-low cycle stress frequency
$∆\sigma_{H}$	High cycle stress range
$\sigma_{a,HCF}$	High cycle stress amplitude
$f_{H}$	High cycle stress frequency
$n_{i}$	Number of loading cycles at the stress level $\sigma_{i}$
D	Cumulative damage
$N_{HCF,i}$	Number of cycles to failure of HCF
$q_{i}$	Damage exponent
$D_{c}$	Coupled damage
$N_{fp}$	Model predicted life
$P_{error}$	Model prediction error
α	Ratio of high-low cycle stress amplitude
$∆\sigma_{L}$	Low cycle stress range
$\sigma_{a,LCF}$	Low cycle stress amplitude
$f_{L}$	Low cycle stress frequency
$N_{Ri}$	Number of cycles to failure at the stress level $\sigma_{i}$
$n_{HCF,i}$	Number of loading cycles of HCF
$N_{LCF,i}$	Number of cycles to failure of LCF
B, β, γ	Material constants
$N_{B}$	Number of combined cycle blocks
$N_{ft}$	Experimental life
